# Clinical study on postoperative triple-negative breast cancer with Chinese medicine: Study protocol for an observational cohort trial

**DOI:** 10.1097/MD.0000000000011061

**Published:** 2018-06-22

**Authors:** Jiajing Chen, Yuenong Qin, Chenping Sun, Wei Hao, Shuai Zhang, Yi Wang, Juan Chen, Lixin Chen, Yiying Ruan, Sheng Liu

**Affiliations:** a Shanghai University of Traditional Chinese Medicine; b Department of Breast Surgery (Integrated Traditional and Western Medicine), Longhua Hospital affiliated to Shanghai University of Traditional Chinese Medicine, Shanghai, China.

**Keywords:** observational cohort study, protocol, traditional Chinese medicine, triple-negative breast cancer

## Abstract

**Background:**

Breast cancer (BC) poses a tremendous threat to the health of women worldwide, especially triple-negative breast cancers (TNBCs). Currently, the curative effect of traditional Chinese medicine (TCM) has been recognized in more and more people worldwide; however, the specific effect has not been systematically evaluated. The purpose of this cohort study is to evaluate the clinical effects of TCM syndrome differentiation on recurrence and metastasis rate, survival rate, and the quality of life in patients with TNBC.

**Methods:**

This study is a multicenter observational cohort trial taking 2 years. A total of 620 patients will be allocated at a ratio of 1:1 to receive TCM or not. The primary outcomes are progression-free survival (PFS) and overall survival (OS), which are calculated at the end of the trial. Secondary outcomes include TCM symptoms, Karnofsky Performance Status (KPS), ECOG score, European Organization for Research and Treatment of Cancer (EORTC) Breast-Cancer-Specific Quality of Life Questionnaire (EORTC QLQ-BR23), as well as clinical indicators including tumor markers, immune function evaluation, chest computed tomography/magnetic resonance imaging, and abdominal B-ultrasound. Assessments will be performed at baseline and 3, 6, 9, 12, 16, and 20 weeks after observation.

**Discussion:**

This will be the first clinical trial to evaluate the PFS and OS in TNBC patients receiving TCM, which may be used to assess the feasibility of a larger-scale clinical trial in the future, and formulate a standardized TCM treatment plan.

**Study registration:**

ClinicalTrials.gov (NCT03332368).

## 1 Background

Breast cancer (BC) is one of the most serious threats to women's health in China, and is the most common causes of cancer death of women in the world.^[1]^ The negative immunohistochemical detection results of estrogen receptor (ER), progesterone receptor (PR), human epidermal growth factor receptor-2 (Her-2) are defined as triple-negative breast cancer (TNBC).^[2]^ Compared with other types of BC, TNBC has a high risk of invasion, local recurrence, and metastasis. Moreover, endocrine therapy and Her-2 target therapy are not suitable for patients with TNBC.^[3,4]^ Meanwhile, due to high cost of biological agents, chemotherapy is still a widespread treatment for most of patients without medical insurance in China. The incidence of TNBC is getting younger and younger.^[5]^ Once the disease progress to the late stage, it is more difficult to control, and the average survival time is only about 2 years.^[6]^

Fortunately, due to the impact of traditional Chinese medicine (TCM) treatment in China, the majority of postoperative TNBC patients have received varying degrees of TCM treatment.^[7,8]^ On the basis of previous multicenter, randomized, double-blind, and controlled research, TCM can improve the quality of life of TNBC patients, as well as regulate immune function,^[9]^ inhibit tumor growth,^[10]^ reduce recurrence and metastasis,^[11]^ and extend the survival time compared to chemotherapy drugs and molecular targeted drugs. In addition, compared with the biological agents, TCM prices are relatively low.^[12]^

TCM treatment has a long history and has been proved to have obvious therapeutic effects. Clinical study has found that the 5-year recurrence and metastasis rate is significantly lower in the TCM treatment group than that in the simple Western control group.^[13]^ In addition, a high dose of TCM Decoction has a similar killing effect on MDA-MB-435 compared with cyclophosphamide; meanwhile, TCM Decoction can reduce the expression of vascular endothelial growth factor (VEGF) and its receptors FlK-1 in BC tumor tissue, indicating that TCM has an important role in anti-tumor and anti-metastasis effects.^[14]^

The purpose of this study is to evaluate whether the intervention of TCM can improve the postoperative TNBC patients with disease-free survival (DFS) and overall survival (OS), and then provide strong evidence that TCM treatment has exact effects and obvious advantages. Meanwhile, this study can establish TNBC patient information database, which may contribute to the correct guideline for TNBC patients with TCM treatment, build harmonious relationship between doctors and patients, increase TNBC patients’ compliance, and enhance the confidence to overcome the disease.

## 2 Methods

### 2.1 Design

**Figure 1:**
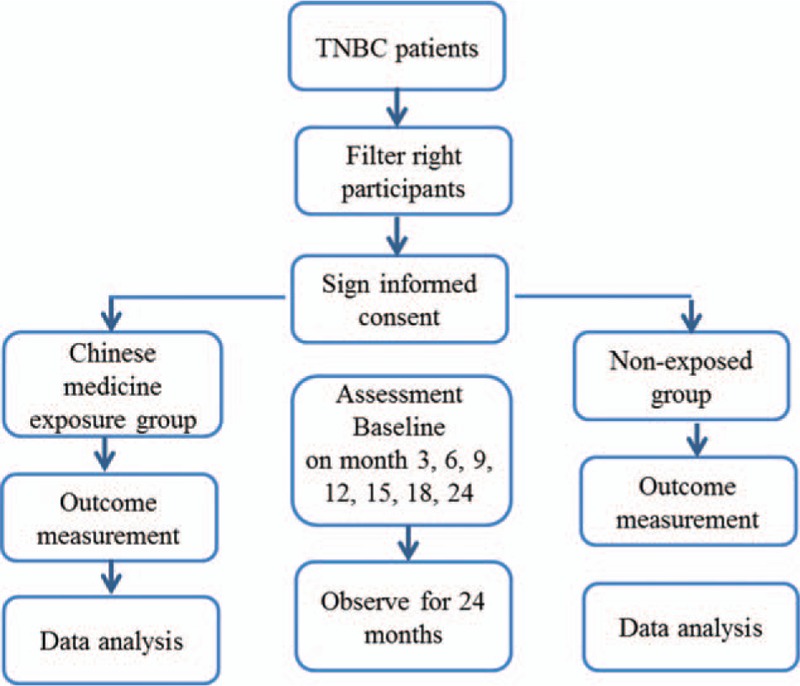
Flow chart of the study.

The study is a multicenter observational cohort trial lasting 2 years in 5 locations, including Longhua Hospital affiliated to Shanghai University of TCM, Shuguang Hospital affiliated to Shanghai University of TCM, Yueyang Hospital of integrated Chinese and Western Medicine, Zhejiang Provincial Hospital of TCM, and Fudan University Shanghai Cancer Center. All the eligible TNBC patients are divided into 2 cohorts: TCM exposure group and nonexposed group, according to whether taking TCM treatment (Fig. 1). Participants in the nonexposed group receive preoperative chemotherapy and/or radiotherapy, as well as postoperative biological agents after surgery, according to the National Comprehensive Cancer Network (NCCN) Breast Cancer Treatment Guidelines. Participants in the TCM exposure group receive TCM combined with Western medicine treatment. TCM treatment is based on syndrome differentiation by inspection, listening and smelling, interrogation, pulse-taking, and palpation, and then provide the most appropriate decoction.

### 2.2 Sample size

Considering the results of the study at MD Anderson Cancer Center in foreign countries^[15]^ showed that the 3-year survival rate of TNBC patients was significantly shorter than that of non-TNBC patients (74% vs 89%, *P* < .01), as well as the lost rate of 20%, the sample size (N) is calculated by the following formula: 
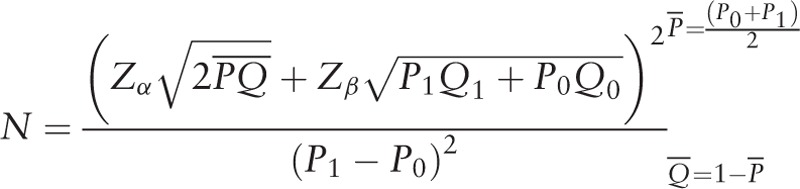


*P*_0_ means the recurrence and metastasis rate of the nonexposed group; *P*_1_ represents the recurrence and metastasis rate of the TCM exposure group, *P*_1_ = RR × *P*_0_, where RR is the relative risk caused by exposure factors.

This study assumes that α = 0.05 and β = 0.10. According to the related study, the rate of recurrence and metastasis was 12% in the TCM group and 33.33% in the western medicine treatment group, respectively. The relative risk (RR) of TCM intervention was that RR = 12%/33.33% = 0.36. Domestic and foreign data showed that the 2-year recurrence and metastasis rate of TNBC patients was 17%, so *P*_0_ = .17, *P*_1_ = RR × *P*_0_ = 0.06.

Substituting the formula, the calculated amount of each group is as follows: 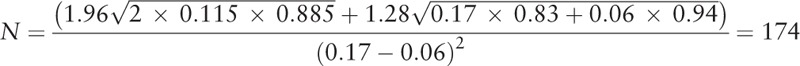


Considering the 20% of drop rate, the confinement factor in different hospitals increased by 10% of the sample size, that is, at least 249 (N ≥ 249) cases should be observed in each group. Then, combined with the study period and the target population distribution of the study unit, 310 cases should be included in the Chinese medicine and Western medicine groups, respectively, in this study.

Before the research, investigators are responsible for the introduction the clinical research related matters and the records of the baseline data, then all participants sign the informed consent form. Patients in the TCM exposure group take Chinese medicine (San Yin Decoction) as well as original Western medicine, while patients in the nonexposed group only receive Western medicine treatment. The study will be performed during 2 years, and all results are recorded according to the Case Report Form (CRF).

### 2.3 Outcomes

#### 2.3.1 Primary outcomes

Primary outcomes include progression-free survival (PFS): the time from treatment to disease recurrence or death due to disease progression; and OS: the time from the beginning of treatment to death due to any cause. These 2 indicators are calculated at the end of the trial.

#### 2.3.2 Secondary outcomes

Secondary outcome are as follows:

1) TCM symptom: based on the “Chinese Medicine New Drug Clinical Research Guidelines (Trial)” (China Medical Science and Technology Press, 2002), the investigators evaluate the TCM symptom every 3 months using scoring method as summarized in Table 1. The efficacy is assessed at 3 levels: significant improvement, the integral value after treatment decreased by ≥ 70% than the integral value before treatment; partial improvement, the integral value after treatment decreased by ≥ 30% than the integral value before treatment; no improvement, before and after treatment without change or after treatment integral value than the integral value before treatment decreased by < 30%. All results are recorded in the CRF table;
2) Karnofsky Performance Status (KPS): the KPS ranking^[16]^ runs from 100 to 0, where 100 is “perfect” health and 0 is death. Practitioners occasionally assign performance scores in between standard intervals of 10. The investigators evaluate the KPS score every 3 months using scoring method as summarized in Table 2. The efficacy is assessed at 3 levels: improved, KPS score increased > 10 points and maintained for more than 4 weeks; stable, KPS score no significant change; worsening, KPS score reduced by < 10 points. All results are recorded in the CRF table;
3) ECOG score: the Eastern Cooperative Oncology Group (ECOG) score (published by Oken et al in 1982),^[17]^ runs from 0 to 5, with 0 denoting perfect health and 5 denoting death. The investigators evaluate the ECOG score every 3 months, and the efficacy is assessed using scoring method as summarized in Table 3. All results are recorded in the CRF table;
4) European Organization for Research and Treatment of Cancer (EORTC) Breast-Cancer-Specific Quality of Life Questionnaire (EORTC QLQ-BR23)^[18]^: This questionnaire focuses on physical condition and the quality of life of all participants. The investigators evaluate the score every 3 months, and the efficacy is assessed using scoring method. All results are recorded in the CRF table;
5) Clinical indicators: Tumor markers (CEA, CA153, CA125, CA199), immune function evaluation (NK, CD3, CD4, CD8, CD4/CD8), chest computed tomography (CT)/magnetic resonance imaging (MRI), and abdominal B-ultrasound are detected to monitor whether there is the tumor recurrence and metastasis. All the participants perform tumor markers check and immune function evaluation every 3 months, as well as chest CT/MRI and ECT every 6 months;
6) Safety indicators are carried out to assess drug safety and whether the participants have any adverse reactions. Blood, urine, and feces routine, liver and kidney function, abdominal B-ultrasound, and electrocardiogram are performed every 3 months. If the investigators find any adverse events (AEs), the event should be assessed according to the WHO acute and subacute side effects of the performance and indexing criteria for assessment. All results are recorded in the CRF table.

**Table 1:**
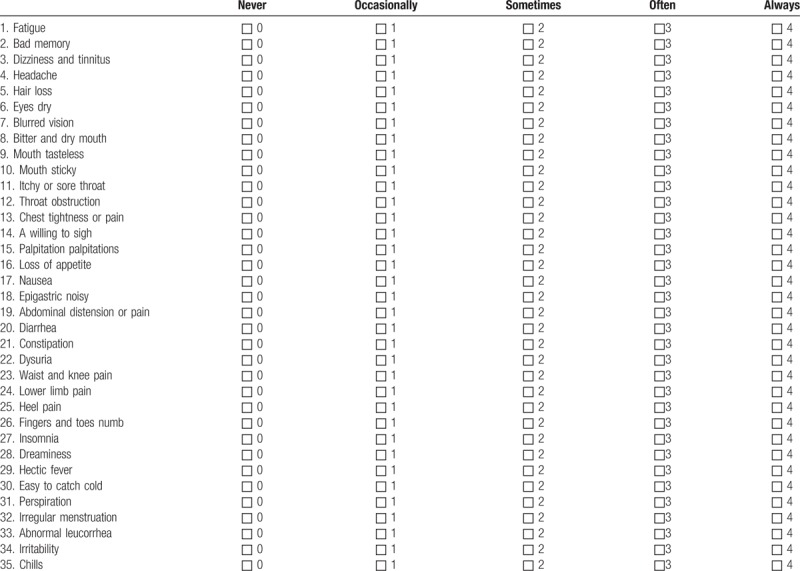
TCM symptom assessment table.

**Table 2:**
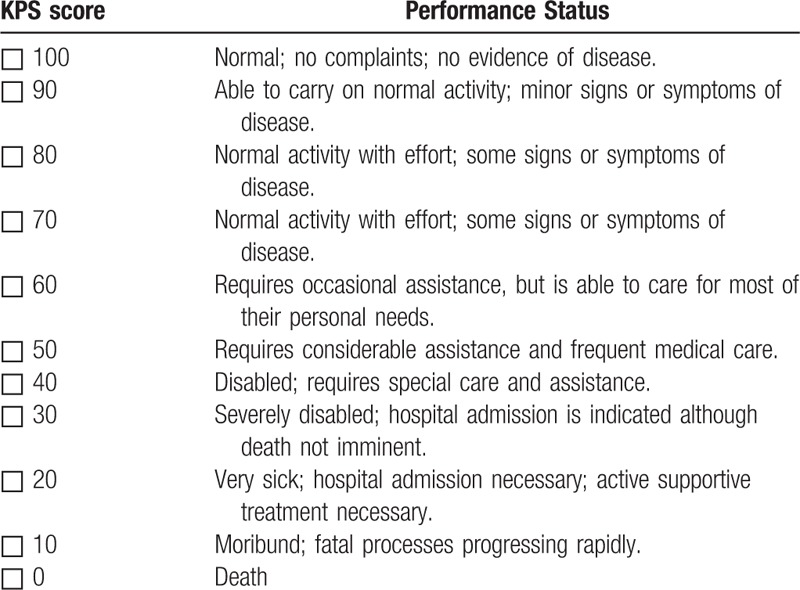
KPS symptom assessment table.

**Table 3:**
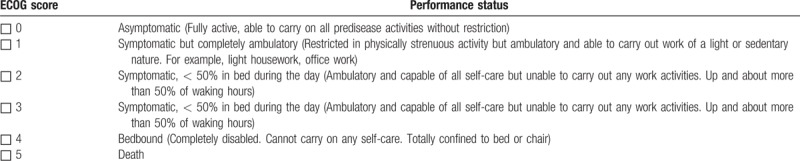
ECOG symptom assessment table.

All the participants are followed by telephone and outpatient visits, and the investigators track the situation of each participant (Table 4).

**Table 4:**
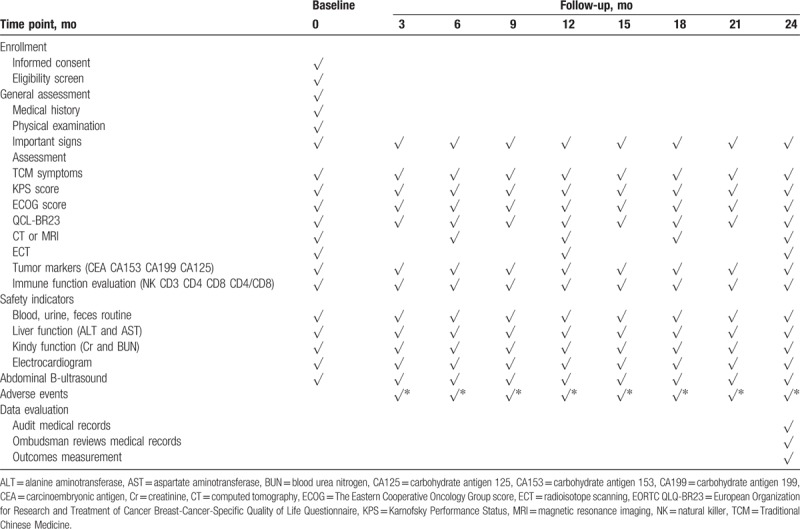
Schedule of enrollment and assessment.

### 2.4 Study participants

All the clinical and pathological diagnoses are defined according to the Chinese Medical Association prepared the “clinical treatment guidelines- tumor volume.” On the basis of the 2002 American Cancer Society “AJCC breast cancer staging standards,” internationally recognized TNM staging, lymph node biopsy, pleural effusion, and other tests were used to confirm the recurrence of metastatic lesions.

### 2.5 Inclusion and exclusion criteria

Participants’ inclusion criteria are as follows: The primary BC after surgical treatment, the pathological diagnosis of breast epithelial tumors (BC), ER, PR, and Her-2 immunohistochemical results were negative; no recurrence of metastasis; KPS score ≥ 60 points; aged 18 to 75 years (including 18 and 75 years); no serious organic or functional disorders, no drugs, and food allergy; and willing to receive treatment, observation, and inspection.

Participants’ exclusion criteria are as follow: do not meet the inclusion criteria; patients expected to survive < 6 months; complicated with cardiovascular and cerebrovascular, liver, kidney, hematopoietic system of serious primary disease, and mental illness; breastfeeding, pregnancy, or women preparing for pregnancy; allergies and allergies to a variety of drugs; and participating in other drug subjects.

### 2.6 Statistical analysis

SPSS 18.0 (SPSS, lnc., Chicago, IL), Stata10.0 (StataCorp, College Station, Texas), and other statistical software are used for data analysis with a 2-sided significance level of 0.05, the point estimate, and 95% confidence interval. If the data obey the normal distribution and the variance is homogeneous, data are expressed as the number of cases (n) and the mean ± standard deviation (SD). Before and after treatment at multiple time points, measurement data using repeated measurement of variance analysis and multiple variance analysis are expressed as median (M), minimum (min), and maximum (max). The Kruskal–Wallis H test is used to compare the data between the 2 groups. The numerical variables are measured at multiple time points before and after treatment, and the generalized estimating equations or mixed effect models are used for the classification and sorting variables. If the analysis index is disordered using χ^2^ test, the analysis index is ordered with rank sum test, the 2-way ordered attribute of different data using linear χ^2^ test, and rank sum test. The survival rate is calculated by Kaplan–Meier method (K-M method), and the survival curve is drawn. Multivariate data are analyzed using multiple linear regression analysis, COX regression analysis, trend testing, and so on.

### 2.7 Adverse events

During the entire study period, AEs will be reported and recorded in the participants’ CRF table. The severity of AEs will be graded as mild, moderate and severe, and their relationship between the study groups will also be assessed by clinical judgement. If an AE requiring hospitalization or causing a medically critical situation occurs, the event will be recorded as a severe adverse event (SAE). All SAEs will be reported to the investigator of the attending hospital and the IRB of reference within 24 h after the information has been collected. AEs that cannot deny a relationship to the study must be followed until the AEs have been resolved. Whenever AEs progress to the level of SAEs, the events must also be reported according to the above protocol. At the time of the study result submission, AEs and their relationship to the study will be documented in a table and submitted.

## 3 Discussion

TNBC has a huge population of patients in China; therefore, the effective measures for the prevention and treatment of BC have become the focus of many scholars.^[19]^ Although the current treatment options for BC is chemotherapy in China,^[20]^ TCM treatment has been considered to play an important role in the treatment for BC. In China, many postoperative TNBC patients receive TCM treatment and get good treatment effect. However, few systematic studies on TCM treatment for postoperative TNBC patients have been conducted, which may lead to the failure of TCM treatment due to lacking of treatment norms and compliance, losing the confidence to overcome the disease.

On the basis of the development of evidence-based medicine and the importance of comprehensive treatment of BC,^[21]^ Cancer Treatment Model, including Chinese medicine, surgery, radiotherapy and chemotherapy, endocrine therapy and biological immunization, and other standardized, individualized comprehensive treatment has become the current international trend of cancer treatment, especially BC treatment. This study will verify this model and introduce evidence-based medicine into the field of research, which will be beneficial to the objective evaluation of TCM syndrome differentiation in the treatment of refractory BC and promote the TCM treatment globalization.

After the completion of this study, the database of TNBC patients in China will be established to conduct long-term dynamic follow-up, health education, and medication guidance for patients, as well as change the 1-way and imperative diagnosis, and treatment process in the past. A dynamic interactive process can be established by basic and clinical researchers, which may lead to an active medical treatment model.

We believe that this cohort study will not only prove the curative effect of TCM but also propose a more standardized and humane treatment plan for TNBC people, which will be beneficial to more and more patients through long-term inheritance.

## 4 Trial status

The basic observational cohort clinical study has received governance approval and is registered at ClinicalTrials.gov (NCT03332368). The trial started recruitment in January 2017.

## Author contributions

JJ-C and SL participated in the design and coordination of the study. SL and YN-Q managed funding of the study; WH drafted the manuscript; JJ-C and YW participated in the design of the study and helped to draft the manuscript; WH and SZ participated in the design of the study and helped to design the analysis plan; CP-S and WH participated in the design of the study and helped to write the funding application; JC and LXC and YYR participated in the design and coordination of the study. All authors read and approved the final manuscript.

**Conceptualization:** Jiajing Chen.

**Data curation:** Yuenong Qin, Yi Wang.

**Funding acquisition:** Sheng Liu.

**Investigation:** Yuenong Qin, Shuai Zhang, Yiying Ruan.

**Methodology:** Chenping Sun, Shuai Zhang, Yi Wang, Lixin Chen.

**Project administration:** Wei Hao, Juan Chen.

**Software:** Lixin Chen.

**Supervision:** Sheng Liu.

**Validation:** Wei Hao, Sheng Liu.

**Visualization:** Juan Chen.

**Writing – original draft:** Jiajing Chen.

**Writing – review & editing:** Sheng Liu.
